# That's disturbing! An exploration of the bacteriophage biology of change

**DOI:** 10.3389/fmicb.2013.00295

**Published:** 2013-11-14

**Authors:** Heather K. Allen, Stephen T. Abedon

**Affiliations:** ^1^Agricultural Research Service, United States Department of Agriculture, National Animal Disease Center, Ames, IA, USA; ^2^Department of Microbiology, The Ohio State University, Mansfield, OH, USA

**Keywords:** virus ecology, ecological disturbance, phage-bacteria interactions

The nature of life is change. Organisms change developmentally, morphologically, and physiologically, and they also modify their environments in the process. Such change can be gradual, abrupt, or even imperceptible components of steady states. Temporally, change can range from deterministic and regular processes to stochastic and unusual events. Unusual changes can result from relatively frequent occurrences, such as particularly devastating storms or instead highly uncommon events such as volcanic eruptions, ice ages, and asteroid impacts.

The scale of environmental change can be local or global, seasonal or climatic. The change can be caused by organisms, including as incidental consequences of their activities, such as our own anthropogenic influences on ecosystems and climate. Responses of organisms to change ranges from behavioral to physiological to developmental, and can occur over ecological as well as evolutionary time scales. The study of ecologically relevant physiological responses is described as physiological ecology, or ecophysiology, while evolutionary responses represent Darwinian evolution.

Among effectors of change are parasites, including viruses. In considering the viruses of bacteria—bacteriophages or phages—change to hosts can vary from devastating lytic infections to simple genetic modification via lysogeny. In between are phages that are released from bacteria chronically, with productively infected bacteria continuing to replicate. Phage-induced change also can range from seemingly cosmetic chromatin rearrangements, as triggered by phage T2 during infection of *Escherichia coli* (Murray et al., 1950) to lysogenic conversion as can result in phage-encoded augmentation of bacterial pathogenesis (Addy et al., 2012). Bacteria also can change in response to phage infection, becoming immune to infecting phages by producing virus-specific interfering RNAs that are associated with CRISPR/Cas systems (Richter et al., 2012).

Change within the context of phages themselves is often more subtle. Phage virions can diffuse or be moved between microenvironments or ecosystems, resulting in changes in abiotic conditions. These environmental changes can cause physiological changes to their adsorption abilities (Conley and Wood, 1975). Phage physiology also changes dramatically as phages transition from their virion or free state to that of infecting bacteria, and then again from their phage-infecting form back to the free virion state. These transitions sometimes include a state of quiescence called pseudolysogeny. These pseudolysogenic states can be brought about by environmental conditions, such as bacterial starvation, and might help to promote phage survival (Ripp and Miller, 1997).

**Figure 1:**
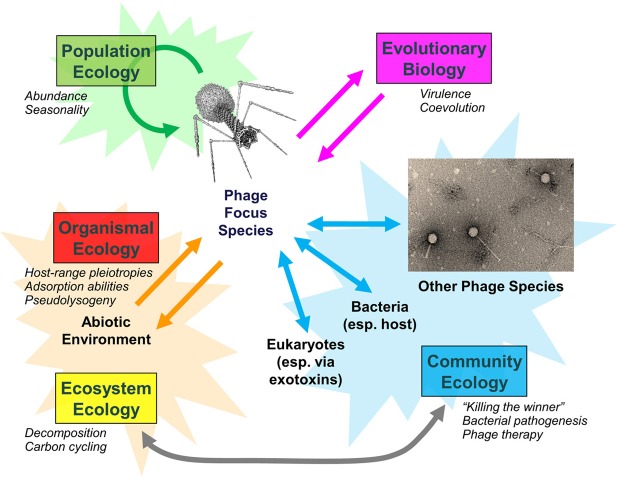
**Bacteriophage population, community, and ecosystem impacts.** Examples of phage-associated changes are italicized. Adapted from Abedon (2009).

Examination of populations, communities, and entire ecosystems reveals that phages play integral roles in both eliciting and responding to disturbances (Figure 1). Phage biology and the relative impact of phages on bacterial populations can change, particularly as phage densities increase from those causing lower versus higher multiplicities of infection (Abedon, 2012). Environmental change in turn can impact viral population densities, including in terms of antibiotic induction of prophages (Allen et al., 2011). Bacterial fitness can change not just with phage quantity but also with phage quality, with greater bacterial fitness costs potentially associated with bacterial evolutionary responses to predation by multiple versus individual phage types (Koskella et al., 2012).

Selection on phages acts primarily on host acquisition, on rates of progeny production, and on survival until host acquisition again becomes a possibility. Suggesting an existence of tradeoffs associated with the optimization of these organismal properties, phage infection strategies may change in their effectiveness, pleiotropically, as phages change from infecting one bacterial strain to another (Duffy et al., 2006). Changes in the abundances of phages and other viruses in complex communities can occur seasonally in estuarian habitats (Winget et al., 2011) or in halophilic viral communities in response to environmental stressors (Santos et al., 2011). Phage abundance also can vary as a function of bacterial abundance, and in turn the cost to bacteria of phage sensitivity can increase as a function of phage abundance. Bacterial existence at high densities thus can result in phage-induced catastrophic changes in bacterial densities, a phenomenon that has been dubbed, “Killing the winner” (Rodriguez-Valera et al., 2009).

On the level of ecosystems, phages can be key contributors to the mineralization of nutrients as they solubilize host bacteria via lysis. As such they contribute to the primary ecological process of soils, that of decomposition and decay. In aquatic environments, phages potentially impact global carbon cycling by short circuiting the movement of carbon and energy to heterotrophic bacteria rather than from cyanobacteria to consumer eukaryotes (Wilhelm and Suttle, 1999). In addition, phages can be added deliberately to environments to motivate change, as seen with phage-mediated biocontrol or phage therapy.

Change thus represents an ongoing and intrinsic aspect of phage biology, with phages both affecting and effecting organismal- population-, community-, ecosystem-, and even global environmental change. In this Research Topic we consider especially the impact of environmental change on communities and ecosystems as those changes may be propagated through phages, gene transfer agents, and viruses of other microorganisms.
